# A modified version of the interlocking finger test as a bedside screening test for visuospatial deficits and dementia in Parkinson's disease

**DOI:** 10.1002/brb3.2516

**Published:** 2022-03-07

**Authors:** Nele Schmidt, Tim Strohmaier, Karsten Witt

**Affiliations:** ^1^ Department of Neurology University Oldenburg Oldenburg Germany; ^2^ Förde Praxis Schönberger Straße 166 Kiel Germany; ^3^ Research Center of Neurosensory Sciences University Oldenburg Oldenburg Germany

**Keywords:** dual syndrome hypothesis, Interlocking Finger Test, Parkinson's disease, Parkinson's disease dementia, screening test

## Abstract

**Introduction:**

Objective of this study was to examine if the Interlocking Finger Test (ILFT) is a suitable bedside screening test for visuospatial functions and/or dementia in Parkinson's disease (PD) patients aiming to facilitate the diagnosis of a dementia syndrome associated with posterior cortical and temporal lobe dysfunction according to the dual syndrome hypothesis (frontostriatal vs. posterior cortical cognitive impairment).

**Methods:**

Forty‐seven PD patients were assessed with the ILFT and an extensive cognitive test battery. The ILFT was carried out in the original version as well as in three modified versions of the test including a fifth figure and/or a more complex rating system, leading to four different ILFT scores (named after the maximum achievable scoring result: ILFT 4, ILFT 5, ILFT 12, and ILFT 15). We conducted a correlation analysis to reveal associations between the ILFT scores and cognitive as well as motor impairments. Receiver operating curve (ROC) analyses were calculated to evaluate the ability of the ILFT scores to predict visuospatial impairments and dementia.

**Results:**

ILFT scores correlated significantly with global cognition, visuospatial functions, memory, attention, and age (*p* < .0125) but not with executive functions, language, education, depression, and motor impairment. The ROC analyses revealed ILFT 15 as best predictor for visuospatial deficits and dementia with an area under the curve of .82 and .88, respectively.

**Conclusion:**

The ILFT is suitable for detecting symptoms of the posterior cortical degeneration syndrome according to the dual syndrome hypothesis. We recommend the use of the modified test version ILFT 15.

## Graphical abstract

The dual syndrome hypothesis differentiates between dopamine modulated frontal‐striatal network dysfunction and a dementia syndrome associated with more posterior cortical degeneration and prodromal visuospatial deficits in Parkinson's disease. In this study, we showed that the Interlocking Finger Test is a suitable bedside screening test for the diagnosis of the posterior cortical degeneration syndrome.

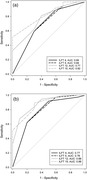

## 1 INTRODUCTION

Cognitive impairment is a common nonmotor symptom in Parkinson's disease (PD). The prevalence of mild cognitive impairment in PD is 40% (Baiano et al., 2020) and the prevalence of dementia in PD is 24–31% (Aarsland et al., 2005). Besides executive dysfunctions and impairments in memory and attention, PD patients often show visuospatial deficits (Aarsland et al., 2010; Curtis et al., 2019; Fernandez‐Baizan et al., 2020; Muslimovic et al., 2005) including impairments in visuospatial perception, orientation, or construction. Deficits in visuospatial abilities increase in the course of the disease (Muslimović et al., 2007) and initially more severe impairments are predictive for the progression of cognitive impairment in PD (Stepkina et al., 2010). Furthermore, visuospatial deficits not only discriminate patients with mild cognitive impairment from those with dementia (Biundo et al., 2014) but are also of prognostic importance regarding the later conversion to development of PD dementia. It was shown that early deficits in posterior cortically based cognitive (e.g., visuoconstructive) tasks that are associated with Lewy body deposition in these areas lead to subsequent dementia while cognitive deficits that are associated with a dopamine modulated frontal–striatal network dysfunction (e.g., executive functions) do not (Williams‐Gray et al., 2007; Williams‐Gray et al., 2009). Based on these and other study results on longitudinal cognitive impairment patterns, Kehagia et al. (2013) proposed the dual syndrome hypothesis which differentiates between two different, partly overlapping syndromes: (1) a dopamine modulated frontal‐striatal network dysfunction in nondemented PD patients which is present at early disease stages and leads to executive and working memory impairments and (2) a dementia syndrome associated with more posterior cortical degeneration, temporal lobe dysfunction, and cholinergic loss characterized by prodromal visuospatial and semantic fluency deficits.

Distinguishing between these two cognitive syndromes in PD at an early disease stage is important to identify specific cognitive risk profiles, especially with regard to different treatment options. While frontostriatal dysfunctions can be improved by dopaminergic treatment (although they are susceptible to overdosing effects), patients with more posterior cortical deficits can benefit from cholinergic treatment (Kehagia et al., 2013). Various tests are available for diagnosing visuospatial deficits which are highly sensitive to the posterior cortical syndrome, however, upper limb motor impairments, tremor, impaired vision, or bedriddenness can be challenging for the neuropsychological diagnostic procedure in PD patients. To our knowledge, a validated bedside screening test for visuospatial deficits is not available so far. The Interlocking Finger Test (ILFT) by Moo et al. (2003) was developed as a screening for parietal lobe dysfunction and was used to detect bimanual apraxia in patients with Alzheimer's disease (Sanin & Benke, 2017). The test consists of four nonsymbolic bimanual gestures which are demonstrated by the examiner and the subject must imitate these gestures. The authors found significant correlations between the ILFT and visuospatial tests (Clock Drawing, Rey‐Osterrieth Complex Figure Test) and showed that the ILFT can predict parietal lobe dysfunction with a good sensitivity and a moderate specificity in a heterogeneous patient group (Moo et al., 2003). In PD patients, the ILFT correlated significantly with visuospatial functions (Clock Drawing Test) as well as with other cognitive domains (e.g., executive functions, memory), and was able to discriminate patients with dementia from those without it with a good specificity and a moderate sensitivity (Souza et al., 2016). In this study, we examined whether the ILFT is a suitable bedside screening test for visuospatial functions and/or dementia aiming to facilitate the diagnosis of the posterior cortical cognitive syndrome in PD. Furthermore, we examined if the predictive ability of the ILFT can be improved by modifying the rating system and adding an additional figure as the original test version has a small scoring range of only 0 to 4 points.

## 2 METHODS

### 2.1 Patients

Forty‐seven patients with PD diagnosed according to the UK Parkinson's Disease Society Brain Bank criteria (Hughes et al., 1992) were included in the analyses. Exclusion criteria were any neurological disorder other than PD and deep brain stimulation. The study was approved by the local ethics committee. All procedures contributing to this work comply with the ethical standards of the relevant national and institutional committees on human experimentation and with the Helsinki Declaration of 1975, as revised in 2013. All participants gave their informed consent to participate in the study in written form.

### 2.2 Interlocking Finger Test

All patients executed the ILFT. In this test, the investigator demonstrates consecutively nonsymbolic bimanual gestures, and the participants are asked to imitate these figures, one at a time, as accurate as possible. For subsequent evaluation, photos of the finished hand positions were taken. The original version of the ILFT includes four figures. They are scored with one point for each correctly imitated interlocking finger component of the figure regardless of the noninterlocking fingers or posture of the arms. In our study, we made two modifications of the ILFT: (1) a fifth figure was added (all figures are shown in the Figure S1) and (2) a more complex rating system was developed. In the modified rating system, three points were given for each figure. One point was given when the interlocking finger component including all fingers which are directly interlocked with the fingers of the other hand was imitated accurately. Therefore, the first point corresponds with the original test score by Moo et al. (2003). The second point was given when the noninterlocking fingers were placed correctly. The third point was given when both hands were orientated correctly to each other and to the participant's body irrespective of the individual fingers. The score for each figure was therefore ranging from 0 to 3. According to these modifications, four different test scores were calculated, named after the maximum achievable scoring result: ILFT 4 (4 figures, original one‐point scoring system), ILFT 5 (5 figures, original one‐point scoring system), ILFT 12 (4 figures, modified three‐point scoring system), and ILFT 15 (5 figures, modified three‐point scoring system).

### 2.3 Cognitive functioning and clinical data

We used the Mini Mental State Examination (MMSE; Folstein et al., 1975) and the Parkinson Neuropsychometric Dementia Assessment (PANDA; Kalbe et al., 2008) as screening instruments for global cognitive functioning. Furthermore, we conducted an extensive neuropsychological test battery covering the following domains:

- Visuospatial functions (Consortium to Establish a Registry for Alzheimer's Disease/CERAD, Morris et al., 1989: Constructional praxis copy; Leistungsprüfsystem 50+, Sturm et al., 1993: Mental rotation and Spatial sense),
- Executive functions (CERAD: lexical and phonemic fluency tests, Trail Making Test B/A; Wechsler Memory Scale—Revised, Härting et al., 2000: Digit span reversed; Modified Card Sorting Test, Nelson, 1976: categories completed and perseverative errors),
- Attention (Brief Test of Attention, Schretlen, 1997; Stroop Test, Bäumler, 1985: reaction time),
- Memory (CERAD: Word list Learning and Recall, Constructional praxis recall), and
- Language (CERAD: Boston Naming Test).

Based on these tests, the patients were classified into PD with and without visuospatial impairments, and with and without dementia, respectively. Visuospatial impairment was diagnosed if a patient scored ≥ 1.5 standard deviations below normative data in at least one test assigned to the visuospatial domain. Dementia was diagnosed according to the Movement Disorder Society Task Force criteria (Emre et al., 2007) including (1) cognitive test scores ≥ 1.5 standard deviations below normative data in at least two different cognitive domains, (2) cognitive decline reported by the patient or a relative, and (3) significant impairment in activities of daily living.

Disease severity was rated with the Unified Parkinson's disease rating scale motor score (UPDRS III; Fahn et al., 1987). The short form of the Geriatric Depression Scale (GDS; Sheikh & Yesavage, 1986) was used to assess depression. Levodopa equivalent daily dose (LEDD) was calculated according to Tomlinson et al. (2010).

### 2.4 Statistical analyses

Statistical analyses were carried out using SPSS 25 (IBM SPSS Statistics for Windows, IBM Corp., Armonk, NY, USA) and SigmaPlot version 11.0 (Systat Software, Inc., San Jose, CA, USA). Given the fact that none of the ILFT scores were normal distributed according to the Shapiro–Wilk test, we used nonparametric statistical tests. To reveal associations between ILFT scores and sociodemographic, clinical, and neuropsychological data, Spearman's rank correlation coefficients were calculated. As we computed correlations for all 4 ILFT scores, we used Bonferroni correction for multiple testing to decrease the risk of false positive errors. Therefore, effects were considered significant at *p* ≤ .0125 (.05/4). To evaluate the ability of the ILFT scores to predict visuospatial impairments and dementia, receiver operating characteristic (ROC) curve analyses were performed. To evaluate the diagnostic accuracy, sensitivity, specificity as well as positive and negative predictive value (PPV, NPV) were calculated. In addition, the Youden index (Youden, 1950) was computed, defined as the sum of sensitivity and specificity minus 1. To determine the interrater reliability of the ILFT scores, the ILFT rating was carried out by two independent raters (one psychologist and one physician) and Kendall's tau‐*b* coefficient was calculated.

## 3 RESULTS

Twenty‐eight of the PD patients were men and 19 women. Mean age was 66.67 (± 7.61). Disease duration was 7.04 years (± 3.72), UPDRS III score in the medical condition was 22.96 points (± 15.59), LEDD was 775.15 (± 363.21), and GDS score was 4.05 (± 3.40). Mean scores of the ILFT versions were 3.32 for ILFT 4 (± 0.86, range: 1–4), 4.21 for ILFT 5 (± 1.06, range 1–5), 9.32 for ILFT 12 (± 2.00, range: 4–12), and 11.79 for ILFT 15 (± 2.58, range: 5–15). There were no group differences in ILFT scores between men and women (Mann‐Whitney *U* tests, *p* = .612 to .956). All ILFT scores correlated significantly with age (*r* = −.509 to −.590, *p* < .001) but not with education, disease duration, severity of motor symptoms, LEDD, and depression (*p* ≥ .0125). All ILFT scores correlated significantly with at least one global cognition test (*r* = .316, *p* = .039 to *r* = .403, *p* = .007 for the MMSE and *r* = .524, *p* = .001 to *r* = .599, *p* < .001 for the PANDA). Regarding visuospatial test results, all ILFT scores correlated significantly with CERAD Constructional praxis (*r* = .415 to .500, *p* < .001 to *p* = .004) and LPS 50+ Spatial sense (*r* = .456 to .532, *p* < .001 to *p* = .004). Furthermore, there were significant correlations between ILFT results and CERAD memory scores (*r* = .370 to .593, *p* < .001 to .011) as well as Brief Test of Attention (*r* = .392 to .523, *p* < .001 to *p* = .008). Beyond that, the correlation analysis showed only sporadic significant results between isolated ILFT scores and phonematic word fluency and Stroop Test reaction time. All correlations can be seen in Table 1.

**TABLE 1:** Correlations between ILFT scores and sociodemographic, clinical, and cognitive data

	ILFT 4	ILFT 5	ILFT 12	ILFT15
*Sociodemographic variables*				
Age	**−.509 (<.001)**	**−.527 (<.001)**	**−.572 (<.001)**	**−.590 (<.001)**
Years of education	.282 (.054)	.291 (.047)	.238 (.107)	.237 (.109)
*Clinical variables*				
Disease duration	−.025 (.870)	−.035 (.816)	−.067 (.654)	−.054 (.719)
UPDRS III	−.158 (.290)	−.170 (.254)	−.258 (.080)	−.206 (.165)
LEDD (mg)	−.003 (.985)	−.008 (.959)	.031 (.837)	.039 (.795)
GDS	−.331 (.037)	−.320 (.044)	−.360 (.022)	−.251 (.118)
*Global cognitive abilities*				
MMSE	.316 (.039)	.341 (.025)	**.379 (.012)**	**.403 (.007)**
PANDA	**.524 (.001)**	**.541 (<.001)**	**.584 (<.001)**	**.599 (<.001)**
*Visuospatial functions*				
CERAD: CP copy	**.415 (.004)**	**.425 (.003)**	**.419 (.003)**	**.500 (<.001)**
LPS 50+: mental rotation	.190 (.215)	.214 (.164)	.308 (.042)	.340 (.024)
LPS 50+: spatial sense	**.456 (.002)**	**.469 (.001)**	**.532 (<.001)**	**.513 (<.001)**
*Executive functions*				
TMT B/A	.127 (.399)	.150 (.318)	.053 (.727)	.020 (.895)
Digit span reversed	.133 (.374)	.132 (.378)	.173 (.245)	.190 (.202)
CERAD: lexical fluency	.207 (.163)	.226 (.127)	.305 (.037)	.332 (.023)
CERAD: phonematic fluency	.186 (.216)	.205 (.171)	**.379 (.009**)	**.368 (.012)**
MCST: categories completed	.212 (.162)	.238 (.115)	.254 (.093)	.292 (.051)
MCST: perseverative errors	−.227 (.134)	−.242 (.109)	−.304 (.042)	−.324 (.030)
*Memory*				
CERAD: word list learning	**.375 (.009)**	**.400 (.005)**	**.425 (.003)**	**.409 (.004)**
CERAD: word list recall	**.553 (<.001)**	**.569 (<.001)**	**.593 (<.001)**	**.552 (<.001)**
CERAD: CP recall	**.370 (.011)**	**.397 (.006)**	**.422 (.003)**	**.448 (.002)**
*Attention*				
Brief Test of Attention	**.392 (.008)**	**.401 (.006)**	**.523 (<.001)**	**.501 (<.001)**
Stroop Test: reaction time	−.325 (.032)	−.344 (.022)	**−.455 (.002)**	**−.458 (.002)**
*Language*				
CERAD: Boston naming test	.227 (.060)	.314 (.031)	.283 (.054)	.345 (.018)

Data are given as Spearman's rank correlation (*p* value, two‐tailed), significant results on *p* ≤ .0125 are in bold.

ILFT: Interlocking Finger Test; UPDRS: Unified Parkinson's Disease Rating Scale; LEDD: L‐dopa equivalent daily dose; GDS: Geriatric Depression Scale; MMSE: Mini Mental State Examination; PANDA: Parkinson Neuropsychometric Dementia Assessment; CERAD: Consortium to Establish a Registry for Alzheimer's disease; LPS: Leistungsprüfsystem; TMT: Trail Making Test; MCST: Modified Card Sorting Test; CP: Constructional praxis.

**FIGURE 1:**
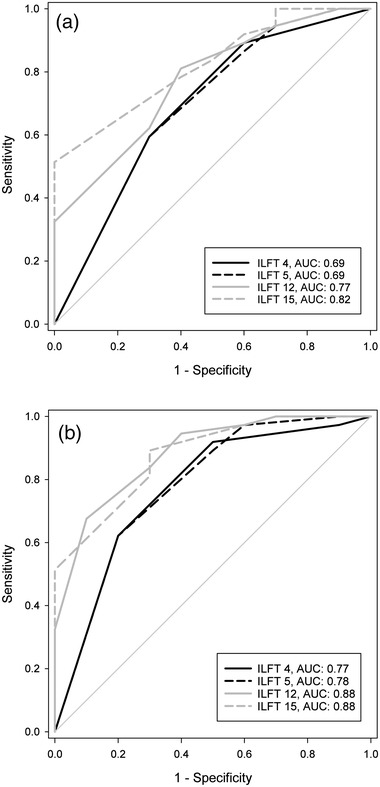
Receiver operating characteristic (ROC) curves of the four Interlocking Finger Test (ILFT) versions. ROC curves demonstrate sensitivity (true positive rate) and 1 − specificity (false positive rate) of the ILFT according to the diagnosis of (a) visuospatial deficits and (b) Parkinson's disease dementia. AUC: area under the curve

The ROC curve analyses using the ILFT scores to predict deficits in visuospatial functions showed that ILFT 15 was the ILFT version with the highest AUC (.82). The AUC significantly different from those of ILFT 4 (*p* = .02) and ILFT 5 (*p* = .02). No significant difference was found between the AUCs of ILFT 15 and ILFT 12 (*p* = .13). Best possible cut‐off score was 12.5 with a Youden index of .51. PPV was .36 and NPV was 1. Regarding dementia, the analyses revealed ILFT 12 and ILFT 15 as best predictors with both an AUC of .88 and maximum Youden index of .58 and .59 at cut‐off scores 9.5 and 10.5, respectively. PPV and NPV were .43 and .96, respectively, for the ILFT 12 and .64 and .92, respectively, for the ILFT 15. The AUC of the ILFT 12 differed significantly to those of ILFT 4 (*p* = .02) and ILFT 5 (*p* = .04) and the AUC of ILFT 15 was significantly different from the AUC of ILFT 4 (*p* = .04). There were no significant differences between the other AUC pairs. All results of the ROC curve analyses can be seen in Table 2 and Figure 1. Interrater reliability ranged from *τ* = .755 to .891 for the four ILFT scores.

**TABLE 2:** Diagnostic values of the receiver operating characteristic curve analyses for the Interlocking Finger Test

	Test	Sens.	Spec.	*Y*	PPV	NPV	Cut‐off value	AUC	SE	95% CI	*p*
**Prediction of visuospatial deficits**	**ILFT 4**	.7	.59	.29	.32	.88	3.5	.69	0.10	0.50 to 0.88	.067
	**ILFT 5**	.7	.59	.29	.32	.88	4.5	.69	0.10	0.50 to 0.89	.063
	**ILFT 12**	.60	.81	.41	.46	.88	8.5	.77	0.08	0.61 to 0.93	.009
	**ILFT 15**	1.00	.51	.51	.36	1.00	12.5	.82	0.06	0.70 to 0.95	.002
**Prediction of dementia**	**ILFT 4**	.80	.62	.42	.36	.92	3.5	.77	0.09	0.60 to 0.94	.009
	**ILFT 5**	.80	.62	.42	.36	.92	4.5	.78	0.09	0.60 to 0.95	.007
	**ILFT 12**	.90	.68	.58	.43	.96	9.5	.88	0.06	0.77 to 0.99	.000
	**ILFT 15**	.7	.89	.59	.64	.92	10.5	.88	0.06	0.77 to 0.99	.000

Sens.: sensitivity; Spec.: specificity; *Y*: Youden's index; PPV: positive predictive value; NPV: negative predictive value; AUC: area under curve; SE: standard error; CI: confidence interval.

## 4 DISCUSSION

We found significant correlations between ILFT scores and global cognition, visuospatial functions, memory, attention, and age but not between ILFT scores and executive functions, language, education, depression, and disease related variables such as disease duration and LEDD. Remarkably, the ILFT did not reflect motor impairment, given the lack of significant correlations between ILFT and motor scores. This specific property classified the ILFT as a cognitive rather than a motor task. The ROC analyses revealed ILFT 12 and ILFT 15 as best predictors for visuospatial deficits and dementia with good negative and moderate positive prediction values.

The correlation analysis showed that the ILFT highly correlated with global cognition, visuospatial functions, memory, and attention. These results are in line with a previous study in which significant correlations with tests of the same neuropsychological domains were found (Souza et al., 2016). We did not find relevant correlations with executive functions what is in line with Moo et al. (2003). As executive functions are characteristic for the dopamine modulated frontal‐striatal network dysfunction syndrome (albeit except for semantic fluency), the results support our hypothesis that the ILFT is sensitive for the posterior cortical degeneration syndrome according to the dual syndrome hypothesis. However, Souza et al. (2016) found correlations between the ILFT and several executive tests what might be due to the high rate of PD patients with a coexistence of a dementia syndrome in their study (40.5% vs. 21.3% in our study). Remarkably, the ILFT did not correlate significantly with any disease related variable, although it is a common problem in clinical praxis that motor impairment affects visuospatial (especially visuoconstructive) test performance in PD. We found negative correlations between ILFT and age, indicating that older people tended to achieve lower ILFT scores. An age‐dependency was also shown by Souza et al. (2016) who supposed that the ILFT is able to detect subtle cognitive changes associated with aging. The result is in accord with the fact that older age is a significant predictor for dementia risk in PD (Williams‐Gray et al., 2009). Moo et al. (2003) did not find significant correlations between ILFT and age; however, only 38 out of 69 patients were included in this calculation what may have biased the results.

The ROC curve analyses showed good negative prediction values for predicting visuospatial deficits and dementia in PD patients with no or only low risk of false negative results, indicating that the ILFT is suitable for the use as a bedside screening test. The positive prediction values of the ILFT were moderate, meaning that there is a higher chance of false positive results. Therefore, patients with a result below the cut‐off score must undergo a formal neuropsychological examination to verify the ILFT result what is in line with the nature of a screening test. The predictive values of our study are comparable to those of previous studies with PD (Souza et al., 2016) and AD patients (Sanin & Benke, 2017). The fact that motor impairments did not affect the ILFT result and its easy implementation with a test duration of a few minutes maximum and no need for test material or equipment (e.g., table, pencil, watch) argue for the ILFT as an appropriate screening instrument. Furthermore, the test showed good interrater reliability.

There were significant improvements in the prediction of visuospatial deficits and dementia when using the modified versions of the ILFT. For predicting visuospatial deficits, ILFT 15 turned out to be the best predictor according to AUC and Youden index. Therefore, the ILFT 15 is recommended as bedside screening test for the diagnosis of visuospatial deficits. At a result of 12 points or lower, an extensive neuropsychological testing should be carried out. Regarding the prediction of PD dementia, ILFT 15 is the best predictor with a slightly higher Youden index as the ILFT 12 which is why we here also recommend the modified version ILFT 15 that contains an additional figure and a more complex rating system than the original. Cut‐off for prediction of PD dementia is 10.5, meaning that a score of 10 or lower entails further diagnostic procedure.

A limitation of the study is that visuospatial functions are a multidimensional construct which could not be completely represented in the cognitive test battery. However, even in a formal neuropsychological testing are often isolated tests used that are not covering the entire spectrum of visuospatial deficits. Furthermore, long‐term studies examining if patients with deficits in the ILFT will develop a dementia syndrome in the course of the disease are necessary to verify our results.

In summary, the ILFT significantly correlated with visuospatial functions, memory, attention, global cognitive abilities, and age indicating that it is suitable for detecting symptoms of the posterior cortical degeneration syndrome according to the dual syndrome hypothesis. We recommend the use of ILFT 15 with cut‐off scores of 12.5 for predicting visuospatial deficits or 10.5 for predicting PD dementia, respectively.

## Supporting information

Figure S1. Interlocking Finger Test

## Data Availability

The data that support the findings of this study are available from the corresponding author upon reasonable request.
