# Supplementary material for: A modified version of the interlocking finger test as a bedside screening test for visuospatial deficits and dementia in Parkinson's disease
Source: Brain Behav. 2022 Mar 7;12(4):e2516. doi: 10.1002/brb3.2516 (PMC9015001; doi:10.1002/brb3.2516)

**Supplementary Figure 1: Interlocking Finger Test**

Figure 1


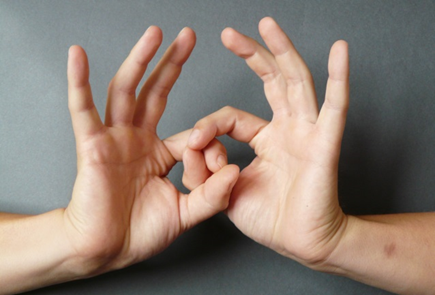


Figure 3


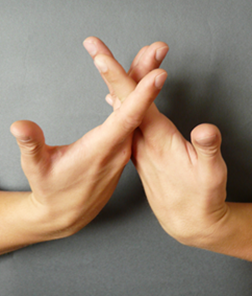


Figure 5 (additional)


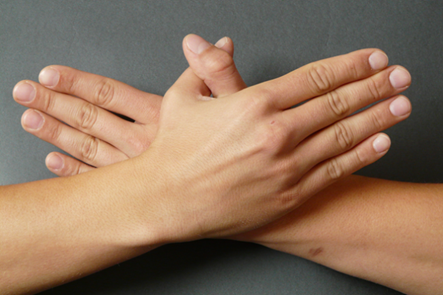


Figure 2


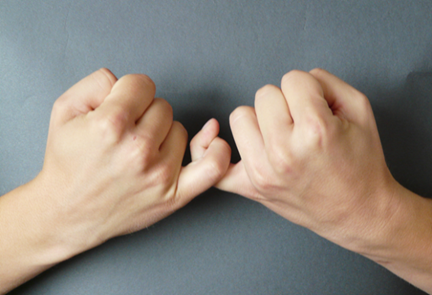


Figure 4


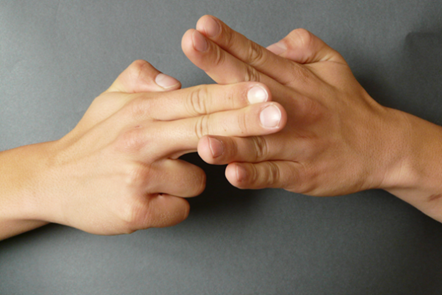

Supplement: Supplementary file 1 — Figure S1. Interlocking Finger Test [file BRB3-12-e2516-s001.docx]
